# A Distributed Approach for Estimating Battery State-Of-Charge in Solar Farms

**DOI:** 10.3390/s19224998

**Published:** 2019-11-16

**Authors:** MCarmen Romero-Ternero, David Oviedo-Olmedo, Alejandro Carrasco, Joaquín Luque

**Affiliations:** Departamento de Tecnología Electrónica, Universidad de Sevilla, Calle San Fernando, 4, 41004 Sevilla, Spain; oviedo@dte.us.es (D.O.-O.); acarrasco@us.es (A.C.); jluque@us.es (J.L.)

**Keywords:** multi-agent system, Bayesian network, expert system, estimation, battery SOC, solar farm, sensors

## Abstract

A common problem in solar farms is to predict when accumulators stop working optimally and start losing efficiency. This paper proposes and describes how to use Bayesian networks together with expert systems to predict this moment by using a telecontrol multiagent system for monitoring solar farms with distributed sensors, which was developed in a previous work. To this end, a Bayesian network model and its implementation are proposed. The resulting system meets the requirements of telecontrol systems (reliability, flexibility, and response time), yields a solution for the prediction of lifespan batteries, and provides the multiagent system with autonomous intelligent capabilities and integrated learning.

## 1. Introduction

The lifespan of a component or device in a power production facility is a parameter that must always be controlled in order to obtain optimum performance in the power station. Furthermore, the health of these devices is crucial for the reliability of the overall system. In the case of solar power plants, one fundamental device is the energy accumulator. Typically, in photovoltaic facilities, the most commonly used type of energy accumulators is lead–acid batteries.

One of the most common problems when using lead–acid batteries involves ascertaining that the performance of the batteries has fallen below recommended levels. There are many studies in the literature that focus on the state of charge of a battery (SOC) [1,2,3,4,5], the majority of which are based on inference systems using dynamic learning [6,7]. The control of the energy output of accumulators [8] or modelling their behavior [9,10] also constitutes the focus of numerous pieces of research. However, specifying when the lifespan of a lead–acid battery has terminated is defined in a parameter that is generally based on the indications given by the manufacturer. However, these indications depend on the operation conditions of the battery and other uncertain parameters [11].

Complex models exist that enable the state-of-health of batteries [12,13] to be ascertained, other models exist that are based on the observation of various internal parameters of the battery itself [11], and yet further models exist that are based on the observation of operation conditions [14] or on intelligent approaches [15,16]. These solutions offer good response reliability but feature a high complexity, which implies specific resources, slow response time, and a longer implementation time, in addition to its inflexibility to inclusion in existing systems.

Some works proposes different strategies for allocating resources and establishing priorities. For example, Conte et al. describe a simulation environment based on a proposed multi-agent system theory for resource management in home automation systems [17,18,19]. Pan et al. propose an energy management strategy to minimize the fuel consumption and the battery charging cost by developing and implementing a battery state-of-charge (SOC) pulse-and-glide strategy [20].

Other works show how a decentralized approach allows to improve estimation and control results. For example, Yang et al. present a framework for the design of collective behaviors for groups of identical mobile agents whose approach is based on decentralized simultaneous estimation and control [21]. Stankovic et al. propose new algorithms for state estimation using a multiagent network [22,23]. Li et al. investigate the average-consensus problem of first-order discrete-time multiagent networks in uncertain communication environments [24]. Menon and Edwards propose a robust fault estimation method for a collection of agents undertaking a shared task and exchanging only relative information over a communication network [25]. Poulakakis et al. describe a collective decision making based on a cooperative multi-agent network [26]. Khalid et al. proposes a combinatorial model involving autoregressive integrated moving average and a nonlinear autoregressive network with exogenous inputs (NARX-net) [27].

Therefore, in order to obtain a control system with time restrictions that is able to handle this complexity in a reasonable and optimal mode, a study was performed to obtain a solution with an efficient and simple reasoning system, which also complied with reliability requirements in the control systems. This study is based on the integration of three technologies: Multiagent systems (MAS), expert systems (ES), and Bayesian networks (BN), with the purpose of fulfilling the requirements of the proposed problem domain: Reliability, scalability, flexibility, optimization, and decision autonomy (intelligent and adaptive).

Multiagent systems enable suitable models to be built for the prediction of complex and dynamic systems in real time [28,29,30]. Bayesian networks provide probabilistic graphic models that allow one to develop systems with reasoning characteristics. This kind of reasoning, in contrast to expert systems, is able to include uncertainty. This uncertainty consists of estimating the probability for unknown variables, based on known variables.

In order to add this reasoning to a multiagent system, a Bayesian model is proposed and developed, which determines the state of each battery, controlled by this multiagent system CARISMA [31], in a probabilistic way. CARISMA already incorporates an expert system for decision-making control. This expert system is responsible for making final decisions based on probabilistic values provided by the newly integrated Bayesian network.

The inclusion of the expert system and its responsibility for its architecture was selected for this problem domain by considering a fundamental point: A system is required whose response is 100% reliable in accordance with the knowledge base that it handles. This allows limits to be set from other inference systems which are not strict in their responses but where supplied information is relevant and must be taken into account.

**Figure 1:**
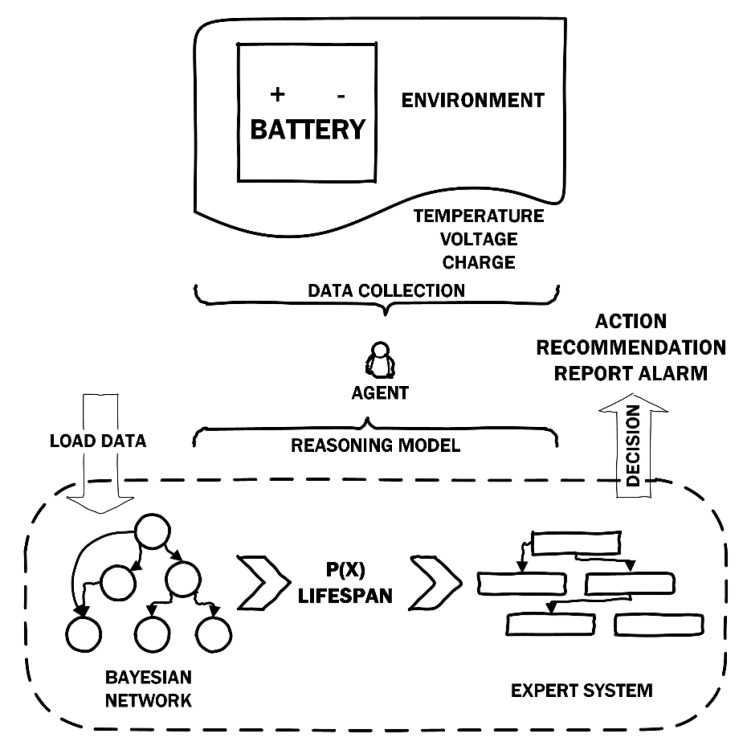
Reasoning model in CARISMA.

Figure 1 shows an example of global system operation. CARISMA has several agents in charge of collecting environment information and acting on it if needed. For the problem domain described in this paper, there are agents in charge of monitoring batteries in a solar farm. These agents implement a reasoning scheme that is shown in Figure 1. This scheme allows the agents to determine the state of the batteries from the collected information (such as battery voltage and temperature, current charge, and number of discharges). This state is represented by a probability value, given by the Bayesian Network.

As shown in Figure 1, the Bayesian network first calculates probabilities of unknown variables and later obtains a probability concerning the lifespan of a battery, which is then passed to the expert system. The expert system then initiates the rules, matching the corresponding action or result based on the value of those variables. Such actions can be, for example, disconnect or redirect energy to another battery, or recommendations and alarms to the teleoperator.

If necessary, probabilistic information provided by the Bayesian network of each agent can be analyzed by other systems outside the expert system. Furthermore, each type of agent has its own Bayesian network and controls the accessible set of variables depending on its level of knowledge.

The model for battery-lifespan prediction is based on a learning model. This uses event patterns and actual sensor data, which include the physical state of the battery. Numerous predictive models are available for battery-lifespan estimation [32,33], but this hybrid model is designed to remove complexity in the system, and incorporates a dedicated and independent inference engine.

The rest of this article is organized as follows: Section 2 describes the new model, Section 3 outlines its implementation, Section 4 shows the measuring and performance checking carried out, and Section 5 provides the conclusions and research.

## 2. Proposed Bayesian Network Model

Modelling a Bayesian network consists of defining its structure (a graph) and its values (tables of probabilities) [34]. To this end, several steps and iterations are usually needed. Since this system has very few variables and states, these steps can be carried out by an expert. Therefore, in this case, the graph and the probability values can be specified manually.

To define this model, a detailed study on solar batteries is performed [35,36]. The specific battery deployed in this hardware system is considered.

### 2.1. System Testing

An accumulation system is employed with six stationary cells SUNLIGHT 2OPzS100-150A (150 A-h C-100). This accumulation system stores energy produced by a solar farm during the hours of sunshine. These lead–acid batteries are the most common type used for solar farms.

### 2.2. Analysis Sensors and Nominal Values of the Accumulation System

Due to the efficient design of the Bayesian model, it is necessary to consider those variables whose values can be ascertained. In this case, the system has the following sensors:

- ρ: Specific gravity (g/mL).
- I_BAT_: Current in the accumulation system (A).
- V_BAT_: Voltage in the accumulation system (V).
- T_BAT_: Temperature of the battery/electrolyte.

For each sensor, the following nominal values are used:

- ρcharged (25 °C) = 1.24 (g/mL).
- ρdischarged (25 °C) = 1.10 (g/mL).
- Temperature compensation = –0.0007 (g/mL·°K).
- V_BAT_nominal_ = 12 (V).

### 2.3. Voltage Values in Load Stages

**Figure 2:**
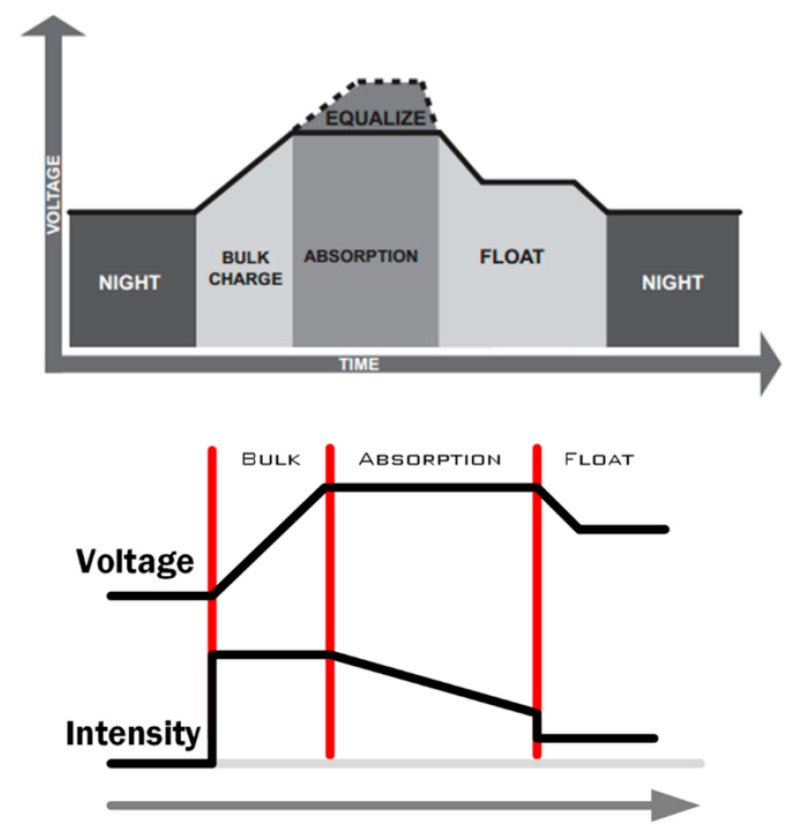
Four-stage battery-charging algorithm (Tristar MPPT Manual).

In order to develop the model, the load stages for this specific model of battery are considered and shown in Figure 2.

As a result, specific voltage values are used:

- V_BAT_bulk_ = 14.40–14.60 (V);
- V_BAT_absorption_ = 14.40–14.60 (V);
- V_BAT_float_ = 13.50 (V);
- V_BAT_equalize_ = 15.30 (V).

### 2.4. Theoretical Basis of the Proposed Model

All concepts in developing the model design are summarized below.

- Battery discharge: When batteries self-discharge, they lose active material from positive plates (lead oxide) and negative plates (lead), which reacts with the sulphuric acid. This reaction reduces specific gravity.
- Battery charge: When batteries are charged, they turn lead sulphate in the terminals into active material and sulphuric acid. This reaction increases specific gravity.
- Battery overload: This is detected when there is a loss of water in the electrolyte and in its evaporation in the form of oxygen and hydrogen (battery gassing).
- Mean depth of discharge: If the discharge is moderate and a great depth is only occasionally reached, then the amount of charge/discharge cycles the battery can support is high. The maximum discharge depth should not exceed 80% of the nominal capacity of the battery.
- Temperature influence on battery lifetime: If the temperature is excessively high, then the chemical reaction over-accelerates in the accumulator and lifetime is therefore reduced. If the temperature is low, lifetime increases, but if it drops too low then there is a risk of freezing.
- Temperature influence on battery capacity: Within limits, an increase in temperature causes chemical process activity to grow, and results in an increase of battery capacity. However, at low temperatures, chemical activity falls, and capacity decreases considerably.
- Charge/discharge history influence on battery capacity: If a battery remains less than fully charged over a long period, then a memory effect prevents the battery from reaching its nominal capacity, and many charge/discharge cycles are needed for its recovery. Battery aging progressively reduces capacity depending on the charge/discharge service.

### 2.5. Bayesian Network Model

In order to develop the Bayesian network model, the following variables are considered in the model:

- Variables with specific evidence: Battery temperature (TBAT, °C), battery output voltage (VBAT, V), and battery charge current (IBAT, A).
- Variables with partial evidence:
  - *Electrolyte density*: This indicates the probability of specific gravity reaching high, medium, or low levels.
  - *Charge level*: This indicates the probability of the battery charge level reaching maximum, high, low, or critical levels.
  - *Discharge depth*: This indicates the probability of discharge depth being low, high, or critical.
  - *Self-discharge*: The probability indicated by this variable refers to the self-discharge rate in the case where the battery has not been used.
  - *Overload*: This indicates the probability of the battery suffering an overload.
  - *Over-discharge*: This indicates the probability of the battery suffering an over-discharge.
  - *Discharge cycle*: This indicates the probability of a whole discharge cycle occurring in the battery.
- Quantifiers: Number of discharge cycles. It is assumed that a complete discharge cycle occurs when the discharge depth is greater than 60%. This variable increases by one when the cycle occurs.
- Output variable: Lifespan. This variable indicates the estimated lifespan in accordance with specific probabilities that are calculated by the Bayesian network.

Certain direct relationships between variables considered for the propagation of probabilities through the Bayesian network are shown in Table 1, Table 2 and Table 3.

**Figure 3:**
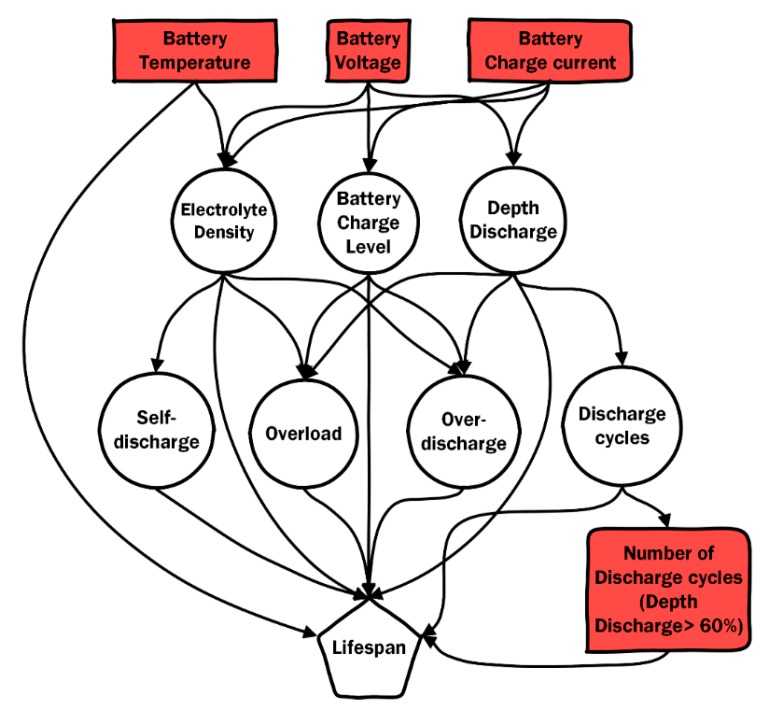
Theoretical Bayesian network model to predict battery lifespan.

Finally, Figure 3 shows the theoretical model proposed for the estimation of battery lifespan in solar farms. It is a dependence network where an A → B connection indicates direct dependence or relevance between variables. This connection indicates that B depends on A, or that A is the cause of B and B is the effect of A. It is also said that A is a parent (or parent-variable) of B, and B is a child (or child-variable) of A. Although the presence of arcs between nodes codifies essential information about the model represented in the network, it is also worth mentioning that the absence of arcs between nodes provides valuable information since the graph codifies conditional independence.

Serial connections or casual chains represent a set of linearly associated variables that denotes dependence among the variables. In this model, for instance, the variable *Overload* depends on *Specific gravity* and the variable *Lifespan* depends on *Overload*. Therefore, when something is known about *Specific gravity*, it is possible to modify the belief about the state of *Overload*, and this information will spread up to *Lifespan*. However, if evidence about *Battery voltage* is found, then the addition of evidence about *Lifespan* will not change the knowledge about *Charge level* and vice versa. In this case, it is said that *Battery output voltage* and *Lifespan* are conditionally independent, given *Charge level*.

### 2.6. Probability Tables

A base probability set was required for each non-observable variable in the proposed Bayesian network. This set was determined by analyzing the previous data and the relations between the variables. The states and probabilities that were set are specified in the following tables (Table 4, Table 5, Table 6, Table 7, Table 8, Table 9, Table 10 and Table 11).

In Table 4, there are nonsensical intervals, and hence these were removed. Intensity values are fitted in each case by the current controller. In Table 7, there are high and continuous self-discharge rates that decrease battery lifespan. The probability in this table refers to the self-discharge rate level if the battery is not used. In Table 10, it is assumed that a discharge cycle occurs when its probability is higher than 80%. All cases not in Table 11 have PLIFESPAN = 0 since they are insufficient to determine whether the battery lifespan is very low.

## 3. Implementation

This implementation of the proposed Bayesian network was carried out over a previously developed multiagent system, based on JADE [37] and called CARISMA [31,38,39,40]. This multiagent system uses expert systems to make decisions or produce recommendations.

In the following sections, the way in which this implementation was integrated into the existing multiagent system is described.

### 3.1. Software for Implementing the Bayesian Network

An external library called SMILE (structural modelling, inference, and learning engine) [41] is used. This library implements decision methods for Bayesian networks and influence diagrams. An environment ready for this library, called GeNIe [41], is also employed. This integrated development environment (IDE) provides a graphic tool to build Bayesian networks in a simple way, by introducing nodes and their relationships. GeNIe enables models of any size and complexity to be built; the only limitation is that of device memory resources.

Additionally, all models created with this interface can be exported in XML for any application that uses the SMILE library, whatever the programming language. Specifically, the variant developed in Java called jSmile was applied.

### 3.2. Expert System Input

Since the CARISMA system had already been implemented with an expert system developed with Drools [31,42], this was reused in order to undertake the objectives with the Bayesian network. First, as discussed earlier, the initial proposal was to connect the Bayesian network to the expert system so that the system would be capable of generating a response to particular input events.

Such events should be able to change the entries in the Bayesian network so that the probability tree can be recreated. However, since the Bayesian network is implemented by SMILE, this cannot be done directly, and therefore the entries in the network are updated by the expert system. To this end, a set of new rules for the expert system was redefined, as in the following example:

RULE 1: Temperature > 90
when
The ambient temperature exceeds 90 °C
then
Fix Bayesian Network: BatteryTemperature value Over50
end

### 3.3. GeNIe: Creating the Bayesian Network

**Figure 4:**
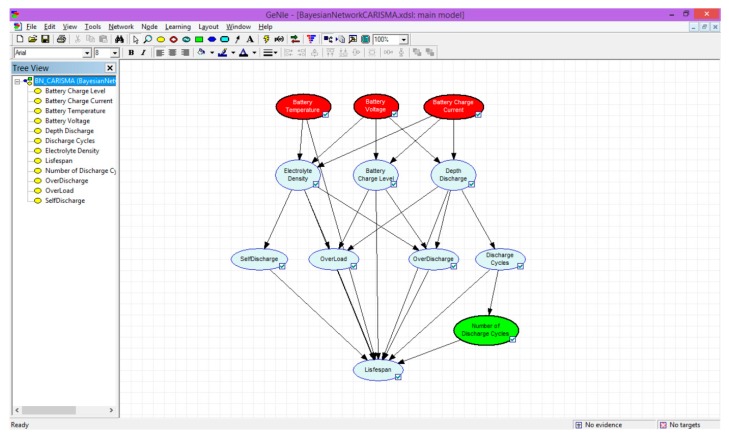
Bayesian network to calculate the battery lifespan in GeNIe.

In order to meet the theoretically set specifications, it was necessary to make certain changes to the model in its implementation. Figure 4 shows the main application window with the Bayesian network developed for this project.

One of the disadvantages of GeNIe software is that a table of discrete values cannot be used, and intervals must be forcibly entered. Thus, five intervals are created in the software that enable, in this case, the probability table to be transferred to the node values.

Another drawback is that the intervals cannot be together in GeNIe intersections, as, for example, in the modelling of one of the more complex nodes, the end node called “Lifespan”. In this case, the probability table contains 5760 values in total. In addition, for each of these cases, there necessarily have to be at least two possible states whose probability sum is exactly 1.

In order to solve this, each node is modelled with one of two alternate states, Good or Bad, referring to the useful life of the battery, which is the ultimate goal of this Bayesian network.

### 3.4. jSmile: Bayesian Networks in Java

Once the Bayesian network has been created by GeNIe, it is then necessary to provide the same information for the inference engine, jSmile. To this end, an XML is properly formatted to jSmile.

Moreover, changes have had to be made in the CARISMA system for its preparation for Bayesian networks. For this purpose, a function called fixedNodeXWithValueY was created which, as its name suggests, allows a state to be attached to a given node. The content of the function is simple, as its operations are very basic. In short, the function indicated carries out the loading of the XML with the Bayesian network, performs the updating of the value of the parameter-node, and asks the implemented expert system, implemented by means of Drools, to trigger those rules corresponding to the final part of inference. The concepts, predicates, and actions necessary for the proper management of knowledge have also been added to the already defined ontology [38]. In this way, the entire system for the creation of interconnected decisions is obtained, as shown in Figure 1.

Finally, a set of rules was created in Drools that is responsible for the prediction of the moment when it is most likely that the remaining battery lifespan becomes very short, and to be sent to the teleoperator agent as a result.

RULE 2: Lifespan bad
when
The probability that lifespan is good is less than 10%
then
Report alarm: Low Lifespan Battery Warning
end

Thanks to this simple rule, when the probability of a good lifespan is less than 10%, or, in other words, when the value of the state Good from the Lifespan node of the Bayesian network is less than 0.1, then the system must inform the teleoperator agent.

## 4. Measurements and Efficiency Tests

### 4.1. Measurements to Be Taken by the System

In order to verify the correct implementation and results thereof, various measurements are taken to meet the required time limits on the specific learning tasks and on the intelligent responses by the inference engine of the Bayesian network. Such measurements include:

- Average time required to recreate the probability tree of the Bayesian network, when a specific event occurs.
- Average time required to generate a response from new information. This includes the processing time of the expert system and the Bayesian network in decision-making.
- Average CPU and memory resources consumed by the Bayesian network.
- Percentage of recommendations and actions by the system that match those expected, partly expected, and those that do not match the expected actions.

### 4.2. Efficiency Tests

In order to study the performance of the implemented system, a specific scenario was set up where its operation was studied in detail. Bear in mind that the implementation was carried out within the base system developed: CARISMA [31,38,39,40]. This system originates from a solar farm that consists of three zones, of which all zones have a common teleoperator agent, although each zone has its own coordinator and operator agents. In addition, each area has its own battery monitored by the system.

**Figure 5:**
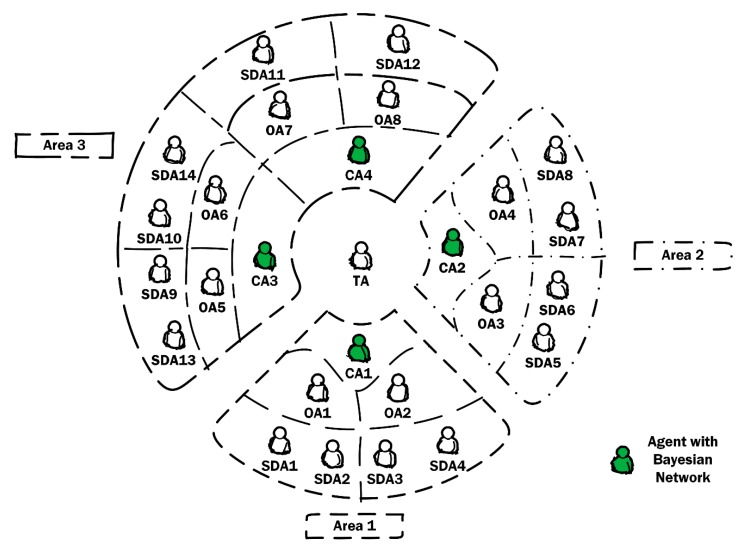
Testing scenario for the CARISMA system.

The problem is shown in Figure 5. The implementation satisfies the following requirements:

- Coordinator agents implement a Bayesian network to perform such monitoring.
- The data needed by the network are obtained by the sensor-dispositive agents (SDA) in the area, which communicate the data to the coordinator agent (CA) for its inclusion in the network. The communication is carried out through the operator agents (OA).
- Final decisions for action based on such monitoring remain the responsibility of the teleoperator agent (TA). The recommendations for actions are supplied to the teleoperator agent by the coordinator agents.
- If more than one coordinator agent coexist in an area, then they may each implement a separate Bayesian network, and their recommendations can be sent to the teleoperator agent, since this is the agent that determines which recommendation should be considered in the case of a contradiction.

The sensor device agents are differentiated according to their functionality: SDA1, SDA5, SDA9, and SDA11 are agents responsible for obtaining the temperature reached by the battery in their respective areas. SDA2, SDA6, SDA9, and SDA11 are agents responsible for measuring the battery voltage and current. The remaining SDAs assume general system functions and can be unrelated to battery functions.

### 4.3. Results

The time required to check the response of the system to the final goal, that is, to obtain an assessment of the battery lifespan controlled by the system, entails a time of no less than two years under real conditions. Therefore, a stand-alone application was developed that changes the value of the various parameters involved, as well as their rate of change, thereby simulating the system performance of two years, within a few hours of simulation.

**Figure 6:**
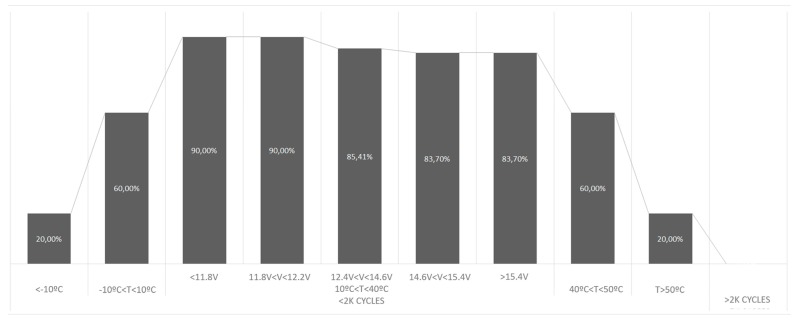
Maximum probability values obtained by the Bayesian network for the CARISMA system.

From this simulation, the average results were obtained as shown in Figure 6 and Table 12 and are understood to represent maximum probability values obtained by the Bayesian network for a given set of circumstances over time.

From the results obtained and then compared with the expected results, as indicated in Section 2 of this article, it is observed that the success rate is high. One can conclude that, at the extremes of tolerance of the battery (>2K cycles, T < –10 °C, T > 50 °C), the system shows over time that there is a high probability that the battery lifespan is about to run out, whereas if we approach the optimum storage conditions (10 °C< T < 40 °C and V < 11.8 V), the probability indicates a greater lifespan. Note that if the probability value of Lifespan is 0, it is considered that the lifespan has ended, while a probability value closer to 1 means better battery status.

A series of tests, designed to test the impact of using Bayesian networks on consumption, has also been performed on the system, at both disk and processor level. Note that these ratings are significant because the system should run even with limited hardware resources. The precision used in the tests is in the range of nanoseconds.

Regarding the CPU consumption, the Bayesian network exerts little impact on this factor. In fact, it is presented that consumption typically ranges between 0.5% and 1% CPU when using the Bayesian network. Regarding the RAM, in no case is the consumption of one megabyte exceeded. It is therefore clear that the impact of the network is negligible.

It should also be borne in mind in tests that, of the three network source nodes, the least expensive in its modification and recalculation of the probability tree is that corresponding to the temperature node, since there is a significant increase in the processing of the two remaining source nodes. Finally, the time required to process a response (message submission or execution of action) by the Bayesian network after having received an input tree was in the area of one millisecond (1 ms).

## 5. Conclusions and Future Work

A complete multiagent system responsible for the monitoring and management of an automated set of solar farms has been developed. The agents of such a system have been equipped with artificial intelligence through a Bayesian network and an expert system. The latter allows the system to control it in an optimized way without any significant increase in resource consumption of the most important elements in a solar panel. Once completed, the system is verified through real testing, and simulations of the responses obtained are adequate, with only insignificant human intervention.

Therefore, the creation of basic distributed intelligence within a multiagent system has been attained, with sufficient capacity to meet its objectives without human intervention. This research demonstrates that it is possible to integrate various inference systems in a multiagent system, which is subject to requirements in time and hardware resources, and to integrate the improvements that this implies.

Regarding the conclusions from the point of view of theory obtained from this research, it can be observed that the combination of inference models and multiagent systems ensures suitable system reliability and time responses in the supervision functions of batteries and of the control system. In general, it can be concluded that it is possible and even feasible to provide agents with intelligent capabilities where time and resource requirements are imposed by control systems. In addition, this type of implementation completes the practical definition of an agent in its theoretical definition. Agents are intelligent and autonomous in practice and are not merely distributed software modules.

If this research is compared to other multiagent systems [43], then artificial intelligence systems (Bayesian network and expert system) are totally integrated in agents, thereby forming the fundamental part of behavior operation (to monitor the environment or to act over it). In fact, agents have no defined behavior, but their actions and monitoring tasks are always dependent on these artificial intelligence systems. This allows an optimization of their tasks. Furthermore, since integration of these AI systems in each agent is decided by the global system designer, we obtain an exclusive flexibility in constructing their reasoning and, in consequence, a global resources optimization.

As stated in other papers [44,45], many energy control microgrid global optimization methods exist. These methods strive towards better performance from the point of view of the described objective in this case study. However, no real scenario with real restrictions is assumed and these methods are limited to theoretical proposals. This research contributes to towards demonstrating that it is possible to implement a distributed system to control and optimize the operation of an energy microgrid in accordance with actual hardware, software, reliability, and response-time requirements.

Regarding future lines of work, we are working in two research areas. The first line of research involves the improvement of forecasting (from a chemistry perspective), and the second involves applying this model in affective computing for cognitive rehabilitation.

In the first case, in order to check the accuracy of the prediction and due to the complexity of determining the real lead–acid battery lifespan, it remains necessary to perform chemical studies on the electrolyte density and precipitation therein to verify that the results obtained correspond to reality. These checks would also allow the adjustment of the developed Bayesian network models, thereby improving their forecasting.

In the second case, affective computing area is a growing research line. Forecasting human behavior could be useful to improve human-centered solutions in many disciplines. Using and extending this model, we propose that emotional intelligence models be applied to support cognitive rehabilitation. The idea involves extending this model so that it supports children therapy by using gamification, by collecting patient emotions, by predicting consequent emotions, and by helping to apply the rehabilitation process. Furthermore, this kind of application can help doctors analyze the evolution of patients and even modify the therapy dynamically.

## Figures and Tables

**Table 1:** Expected variance of percentage of charge of a battery based on its specific gravity and output voltage.

Charge Percentage	Specific Gravity (g/mL)	Voltage
Charge	1.24	14.40 (or higher)
75%	1.20	-
50%	1.16	-
25%	1.13	-
Discharge	1.10	1.85 (or lower)

**Table 2:** Effect of temperature on battery specific gravity.

Temperature °C	Electrolyte Density
43	+0.012
38	+0.008
32	+0.004
27	+0.000
25	+0.000
21	+0.004
16	+0.008
10	+0.012

**Table 3:** Effect of temperature on battery lifespan.

Temperature °C	% Reduction of Lifespan
25	0
30	30
35	50
40	65
45	77
50	87
55	95

**Table 4:** Probabilities and possible states for electrolyte density.

V_BAT_	I_BAT_	T_BAT_	${P_{\mathrm{high}}}_{\rho }$	${P_{\mathrm{medium}}}_{\rho }$	${P_{\mathrm{low}}}_{\rho }$
>15.4 V	>4.8 A	10–40 C	0.9	0.1	0
14.60–15.40 V (Equalization)	3–5 A	10–40 C	0.4	0.6	0
14.60–12.40 V (Bulk/Absorption/Float)	1.5–8.5 A	10–40 C	0.2	0.6	0.2
12.20–11.80 V (Night/Self-Discharge)	1–1.5 A	10–40 C	0	0.6	0.4
<11.80 V (Discharge)	-	10–40 C	0	0.1	0.9
>15.4 V	4.8 A	40–50C	1	0	0
14.60–15.40 V (Equalization)	3–5 A	40–50 C	0.6	0.4	0
14.60–12.40 V (Bulk/Absorption/Float)	1.5–8.5 A	40–50 C	0.3	0.4	0.3
12.20–11.80 V (Night/Self-Discharge)	1–1.5 A	40–50 C	0	0.7	0.3
<11.80 V (Discharge)	-	40–50 C	0	0.3	0.7
>15.4 V	4.8 A	–10 to 10 C	0.8	0.2	0
14.60–15.40 V (Equalization)	3–5 A	–10 to 10 C	0.2	0.6	0.2
14.60–12.40 V (Bulk/Absorption/Float)	1.5–8.5 A	–10 to 10 C	0.1	0.5	0.4
12.20–11.80 V (Night/Self-Discharge)	1–1.5 A	–10 to 10 C	0	0.4	0.6
<11.80 V (Discharge)	-		0	0	1
-	-	>50 C	0.9	0.1	0
-	-	<–10 C	0	0.1	0.9
high_ρ_ = Density values higher than 1.25 g/ml					
medium_ρ_ = Density values between 1.10 and 1.25 g/ml					
low_ρ_ = Density values lower than 1.10 g/ml					

**Table 5:** Probabilities and possible states for charge level.

V_BAT_	I_BAT_	P_MAX–CHARGE_	P_HIGH–CHARGE_	P_LOW–CHARGE_	P_CRITICAL-CHARGE_
>15.40 V	>4.8 A	1	0	0	0
14.40–15.40 V	3–5 A	0.8	0.2	0	0
14.40–12.20 V	1.5–8.5 A	0.2	0.8	0	0
12.20–11.80 V	1–1.5 A	0	0	0.8	0.2
11–11.80 V	-	0	0	0.2	0.8
<11 V	-	0	0	0	1
MAX CHARGE = 90–100%, HIGH CHARGE = 50–90%, LOW CHARGE = 20–50%, CRITICAL CHARGE = 0–20%					

**Table 6:** Probabilities and possible states for discharge depth.

V_BAT_	I_BAT_	P_CRITICAL-DD_	P_HIGH–DD_	P_LOW–DD_
>12.20 V	1.5–8.5 A	0	0	1
12.20–11.80 V (Night/Self-Discharge)	1–1.5 A	0	0.1	0.9
11V–11.8V	-	0.2	0.7	0.1
<11.8V	-	0.7	0.3	0
CRITICAL DD = 60–100%, HIGH DD = 30–60%, LOW DD = 0–30%				

**Table 7:** Probabilities over a high self-discharge rate.

${P_{\mathrm{high}}}_{\rho }$	${P_{\mathrm{high}}}_{\mathrm{self}-\mathrm{discharge}}$
0.8–1	1
0.5–0.8	0.5
0.2–0.5	0.2
0–0.2	0

**Table 8:** Probabilities for overload risk.

P_LOW-DD_	${P_{\mathrm{high}}}_{\rho }$	P_MAX–LOAD_	P_OVERLOAD_
0.8–1	0.8–1	0.8–1	1
0.5–0.8	0.8–1	0.8–1	0.8
0.2–0.5	0.8–1	0.8–1	0.5
0–0.2	0.8–1	0.8–1	0.2
0.8–1	0.5–0.8	0.8–1	0.9
0.5–0.8	0.5–0.8	0.8–1	0.7
0.2–0.5	0.5–0.8	0.8–1	0.4
0–0.2	0.5–0.8	0.8–1	0.1
0.8–1	0.2–0.5	0.8–1	0.5
0.5–0.8	0.2–0.5	0.8–1	0.4
0.2–0.5	0.2–0.5	0.8–1	0.3
0–0.2	0.2–0.5	0.8–1	0.1
0.8–1	0–0.2	0.8–1	0.4
0.5–0.8	0–0.2	0.8–1	0.2
0.2–0.5	0–0.2	0.8–1	0.1
0–0.2	0–0.2	0.8–1	0
0.8–1	0.8–1	0.5–0.8	0.7
0.5–0.8	0.8–1	0.5–0.8	0.4
0.2–0.5	0.8–1	0.5–0.8	0.4
0–0.2	0.8–1	0.5–0.8	0.1
0.8–1	0.5–0.8	0.2–0.5	0.2
0.5–0.8	0.5–0.8	0.2–0.5	0.2
0.2–0.5	0.5–0.8	0.2–0.5	0.2
0–0.2	0.5–0.8	0.2–0.5	0.2
0.8–1	0.2–0.5	0–0.2	0.1
0.5–0.8	0.2–0.5	0–0.2	0.1
0.2–0.5	0.2–0.5	0–0.2	0.1
0–0.2	0.2–0.5	0–0.2	0.1

**Table 9:** Probabilities for over-discharge risk.

P_CRITICAL–DD_	P_LOWρ_	P_CRITICAL-LOAD_	P_OVER-DISCHARGE_
0.8–1	0.8–1	0.8–1	1
0.5–0.8	0.8–1	0.8–1	0.8
0.2–0.5	0.8–1	0.8–1	0.5
0–0.2	0.8–1	0.8–1	0.2
0.8–1	0.5–0.8	0.8–1	0.9
0.5–0.8	0.5–0.8	0.8–1	0.7
0.2–0.5	0.5–0.8	0.8–1	0.4
0–0.2	0.5–0.8	0.8–1	0.1
0.8–1	0.2–0.5	0.8–1	0.5
0.5–0.8	0.2–0.5	0.8–1	0.4
0.2–0.5	0.2–0.5	0.8–1	0.3
0–0.2	0.2–0.5	0.8–1	0.1
0.8–1	0–0.2	0.8–1	0.4
0.5–0.8	0–0.2	0.8–1	0.2
0.2–0.5	0–0.2	0.8–1	0.1
0–0.2	0–0.2	0.8–1	0
0.8–1	0.8–1	0.5–0.8	0.7
0.5–0.8	0.8–1	0.5–0.8	0.4
0.2–0.5	0.8–1	0.5–0.8	0.4
0–0.2	0.8–1	0.5–0.8	0.1
0.8–1	0.5–0.8	0.2–0.5	0.2
0.5–0.8	0.5–0.8	0.2–0.5	0.2
0.2–0.5	0.5–0.8	0.2–0.5	0.2
0–0.2	0.5–0.8	0.2–0.5	0.2
0.8–1	0.2–0.5	0–0.2	0.1
0.5–0.8	0.2–0.5	0–0.2	0.1
0.2–0.5	0.2–0.5	0–0.2	0.1
0–0.2	0.2–0.5	0–0.2	0.1

**Table 10:** Probabilities for occurrence of a discharge cycle.

P_HIGH-DD_	P_CRITICAL-DD_	P_DISCHARGE-CYCLE_
0–0.2	0.8–1	1
0.2–0.5	0.5–0.8	0.9
0–0.2	0.5–0.8	0.8
0.5–0.8	0.2–0.5	0.7
0.2–0.5	0.2–0.5	0.6
0–0.2	0.2–0.5	0.5
0.8–1	0–0.2	0.4
0.5–0.8	0–0.2	0.3
0.2–0.5	0–0.2	0.2
0–0.2	0–0.2	0

**Table 11:** Probabilities and possible states for battery lifespan.

T_BAT_	N_DISCHARGE-CYCLES_	P_CRITICAL-DD_	P_OVERLOAD_	P_OVER-DISCHARGE_	P_HIGH-DD_	P_CRITICAL-LOAD_	${P_{high}}_{\rho }$	P_DISCHARGE-CYCLE_	P_LIFESPAN_
-	>2000	-	-	-	-	-	-	-	1
>55 C	<2000	-	-	-	-	-	-	-	0.8
40–55 C	<2000	-	-	-	-	-	-	-	0.4
10–40 C	<2000	-	-	-	-	-	-	-	0
−10–10 C	<2000	-	-	-	-	-	-	-	0.4
−10 C	<2000	-	-	-	-	-	-	-	0.85
10–40 C	<2000	0.8–1	-	0.8–0.1	0.8–0.1	0.8–0.1	-	0.8–0.1	0.95
10–40 C	<2000	0.5–0.8	-	0.8–0.1	0.8–0.1	0.8–0.1	-	0.8–0.1	0.85
10–40 C	<2000	0.2–0.5	-	0.8–0.1	0.8–0.1	0.8–0.1	-	0.8–0.1	0.6
10–40 C	<2000	0–0.2	-	0.8–0.1	0.8–0.1	0.8–0.1	-	0.8–0.1	0.5
10–40 C	<2000	-	-	0.5–0.8	0.8–0.1	0.8–0.1	-	0.8–0.1	0.7
10–40 C	<2000	-	-	0.2–0.5	0.8–0.1	0.8–0.1	-	0.8–0.1	0.4
10–40 C	<2000	-	-	0–0.2	-	-	-	-	0
10–40 C	<2000	-	0.8–1	-	-	-	0.8–1	-	0.95
10–40 C	<2000	-	0.5–0.8	-	-	-	0.8–1	-	0.85
10–40 C	<2000	-	0.2–0.5	-	-	-	0.8–1	-	0.5
10–40 C	<2000	-	0–0.2	-	-	-	0.8–1	-	0.1
10–40 C	<2000	-	0.8–1	-	-	-	0.5–0.8	-	0.85
10–40 C	<2000	-	0.5–0.8	-	-	-	0.5–0.8	-	0.5
10–40 C	<2000	-	0.2–0.5	-	-	-	0.5–0.8	-	0.2
10–40 C	<2000	-	0–0.2	-	-	-	0.5–0.8	-	0.1
10–40 C	<2000	-	0.8–1	-	-	-	0.2–0.5	-	0.5
10–40 C	<2000	-	0.5–0.8	-	-	-	0.2–0.5	-	0.4
10–40 C	<2000	-	<0.5	-	-	-	0.2–0.5	-	0.2
10–40 C	<2000	-	-	-	-	-	0–0.2	-	0

**Table 12:** Probabilities for overload risk.

Maximum Lifespan	
Conditions	Probability
<2K cycles	
<10 °C	0.2
–10 °C < T < 10 °C	0.6
10 °C < T < 40 °C	
< 11.8 V	
1A < I < 1.5A	0.9
1.5A < I < 3A	0.9
3A < I < 4.8A	0.9
I > 4.8A	0.9
11.8V < V < 12.2V	
1A < I < 1.5A	0.9
1.5A < I < 3A	0.777436364
3A < I < 4.8A	0.837
I > 4.8A	0.837
12.4V < V < 14.6V	
1A < I < 1.5A	0.837
1.5A < I < 3A	0.854143636
3A < I < 4.8A	0.837
I > 4.8A	0.837
14.6V < V < 15.4V	
1A < I < 1.5A	0.520568182
1.5A < I < 3A	0.837
3A < I < 4.8A	0.6312
I > 4.8A	0.837
>15.4V	
1A < I < 1.5A	0.837
1.5A < I < 3A	0.837
3A < I < 4.8A	0.837
I > 4.8A	0.346840909
40 °C < T < 50 °C	0.6
T > 50 °C	0.2
>2K cycles	0
